# Risk Factors for Perioperative Myocardial Infarction/Injury and Mortality Following Surgical Treatment of Proximal Femur Fractures: A Cohort Study

**DOI:** 10.2106/JBJS.OA.25.00164

**Published:** 2025-12-22

**Authors:** Matthias Wittauer, Marc-Antoine Burch, Christian Puelacher, Florian Halbeisen, Martin Clauss, Andreas Marc Müller, Christian Müller, Mario Morgenstern

**Affiliations:** 1 Department of Orthopaedic and Trauma Surgery, University Hospital Basel, Basel, Switzerland; 2 Faculty of Medicine, University of Basel, Basel, Switzerland; 3 Surgical Outcome Research Center, University Hospital Basel, Basel, Switzerland; 4 Center for Musculoskeletal Infections, University Hospital Basel, Basel, Switzerland; 5 Department of Cardiology and Cardiovascular Research Institute Basel, University Hospital Basel, Basel, Switzerland; 6 Department of Internal Medicine III, Cardiology and Angiology, Medical University of Innsbruck, Austria

## Abstract

**Background:**

This study aimed to investigate the incidence of perioperative myocardial infarction/injury (PMI) and mortality and to identify associated risk factors, in patients undergoing surgical treatment for proximal femur fractures (PFFs).

**Methods:**

We performed a post hoc analysis of a prospective cohort study and included consecutive patients undergoing surgery for PFFs (femoral neck, intertrochanteric, or subtrochanteric fractures) at a tertiary center between 2014 and 2018. All patients underwent systematic PMI screening using serial high-sensitivity cardiac troponin T measurements. The primary outcomes were incidence of PMI and all-cause mortality at 1 year. Univariable logistic regression identified risk factors for PMI and mortality.

**Results:**

Among 348 patients, 23% developed PMI. PMI incidence did not differ significantly between arthroplasty and osteosynthesis groups (22.0% vs. 24.0%, p = 0.7). A history of myocardial infarction and hypertension was associated with increased PMI risk. One-year mortality was 17.8% overall and higher in patients with PMI compared with those without (27.5% vs. 14.9%, p = 0.013). Significant risk factors for 1-year mortality included low body mass index, history of atrial fibrillation, low preoperative hemoglobin, and higher anesthesiologists class. No associations were found between PMI or mortality and fracture type, implant type, use of bone cement, or anesthesia type.

**Conclusions:**

PMI is common after surgical treatment of PFFs and is associated with increased mortality. Systematic screening improves detection, enabling optimization of perioperative management. We recommend routine PMI screening in high-risk patients undergoing PFF surgery to reduce adverse outcomes.

**Level of Evidence:**

Level II. See Instructions for Authors for a complete description of levels of evidence.

## Background

Proximal femur fractures (PFFs) represent a significant challenge in orthopaedic practice due to their high morbidity and mortality rates^1-4^. As the global population continues to age, the incidence of hip fractures is projected to nearly double in the next 20 to 30 years^5^, with estimates suggesting that annual cases could reach around 1.8 million by 2050^6^. This anticipated surge in PFFs will place a substantial burden on healthcare and social care systems^7^.

Surgical management is the standard treatment for nearly all PFFs^8^. However, a high prevalence of perioperative complications is recorded, including cardiovascular, pulmonary, thrombotic, and infectious events^9,10^. Frailty, characterized by diminished physiological reserves across multiple organ systems, heightens vulnerability to these complications and contributes to elevated mortality rates^11^.

Perioperative myocardial infarction/injury (PMI) has been recognized as a contributor to mortality after noncardiac surgery in general^12-14^ as well as orthopaedic surgery in particular^15,16^. Since most PMIs present without typical ischemic symptoms, they are often overlooked without systematic screening^17,18^. Previous studies have identified several medical risk factors for PMI—such as coronary artery disease, heart failure, hypertension, chronic kidney disease, and advanced age^19-24^. However, orthopaedic parameters, including factors related to hospitalization, fracture characteristics, and surgical treatment, remain underreported.

The aim of this study was to investigate the incidence of PMI and mortality, along with their associated risk factors—particularly those related to fracture type and treatment modality—in patients undergoing surgical treatment for PFFs using a systematic PMI screening program included in routine clinical practice.

## Methods

### Study Design

This was a post hoc analysis of the prospective observational PMI study conducted at our university hospital. We prospectively included consecutive patients undergoing surgery for PFFs, including femoral neck, intertrochanteric, and subtrochanteric fracture (AO/OTA fracture classification type 31A1-3 and 31B1-3), between October 2014 and July 2018 and who were eligible for the routine institutional PMI screening and response program. Ethical approval for this study was provided by the local ethics committee.

### Patient Selection and Study Population

PMI screening was performed in patients at high cardiovascular risk (defined as age ≥45 years with preexisting coronary, peripheral, or cerebrovascular artery disease, or any patient aged ≥65 years) undergoing orthopaedic surgery with a planned postoperative hospital stay of >24 hours. Plasma concentrations of high-sensitivity cardiac troponin T (hs-cTnT) were measured before surgery and on postoperative days 1 and 2. We excluded patients who were screened despite not meeting the inclusion criteria (age <45 years, <24-hour hospital stay), had their surgery cancelled, had cardiac surgery, or had type 1 myocardial infarction within 14 days before surgery.

All included patients underwent risk assessment using the American Society of Anesthesiologists (ASA) classification and the Revised Cardiac Risk Index^25^. Operative treatment for PFFs followed our institutional protocol: per- and intertrochanteric fractures were treated with osteosynthesis (cephalomedullary nail [CMN] or dynamic hip screw [DHS]), whereas femoral neck fractures were managed with arthroplasty (hemiarthroplasty or total hip arthroplasty [THA]) or osteosynthesis (dynamic hip screw or cannulated screws), based on functional status, bone quality, and fracture displacement.

### End Points

PMI was prospectively defined as an absolute increase of hs-cTnT ≥14 ng/L (the 99th percentile of the assays used) above the preoperative value (or between 2 postoperative values if the preoperative measurement was missing) within 3 days after surgery. PMI was adjudicated independently of symptoms or electrocardiogram (ECG) changes. To determine etiology, PMI was centrally adjudicated by 2 independent experts of the institutional cardiac laboratory based on all clinical information obtained during index hospitalization, including ECG, serial laboratory measurements including hs-cTnT and hemoglobin, monitoring of vital signs in the perioperative and intraoperative period, echocardiography, cardiac stress testing, and coronary angiography, if performed.^26^.

Follow-up was conducted to assess all-cause death at 1 year postoperatively. In case of suspicion of an outcome event, study personnel requested reports from the general practitioners, treating facilities, or death registries to record date of death.

### Statistical Analysis

Descriptive analysis was used to summarize the characteristics of the study population. Continuous variables were reported as medians with ranges, while categorical variables were presented as counts and percentages. Group comparisons were conducted using the Fisher exact test for categorical variables and the Wilcoxon rank-sum test for continuous variables. Survival analysis was performed to compare outcomes between patients with and without PMI. Kaplan-Meier survival curves were generated to visualize the time-to-event data, and the log-rank test was used to statistically compare survival distributions between the 2 groups. To identify potential risk factors of PMI, we performed univariable binary logistic regression. To account for the risk of false positives from multiple comparisons, we adjusted p-values using the Benjamini-Hochberg method. For cases of quasicomplete separation, Firth logistic regression was used to reduce bias in parameter estimation. All statistical analyses were performed using the R statistical software (version 4.3.2, The R Foundation for Statistical Computing).

### Institutional Review Board Approval

This study was approved by the local ethics committee (Trial Registration: NCT02573532).

## Results

We included a total of 348 consecutive patients with a PFF, of which 173 (49.7%) were treated with an arthroplasty and 175 (50.3%) with an osteosynthesis. Among those who underwent arthroplasty, 140 patients (80.9%) received a hemiarthroplasty—of which 136 patients (97.1%) had cemented femoral fixation—while 33 patients received a THA, with cemented fixation used in 12 cases (36.4%). Among patients undergoing osteosynthesis, per- and intertrochanteric fractures were predominantly treated with CMN, while 6 patients received a DHS. In the femoral neck fracture subgroup managed with osteosynthesis, 5 patients were treated with a DHS and 1 with 3 cannulated screws. An overview of the baseline characteristics of the included patients is presented in Table I.

**TABLE I:** Patient Baseline Characteristics

Characteristic	Total		Arthroplasty		Osteosynthesis		
	N	%	N	%	N	%	p
No. of patients	348	100	173	49.7	175	50.3	
Age (median and range)	80	51-95	80	59-95	80	51-94	0.684
Height (median and range)	165.5	140-192	165	145-192	166.5	140-190	0.412
Weight (median and range)	65	35-126	65	39-110	65	35-126	1
Female sex	226	64.94	121	69.94	105	60	0.057
Type of fracture							<0.001
Inter/subtrochanteric	169	48.6	0	0	169	96.6	
Femoral neck	179	51.4	173	100	6	3.4	
American Society of Anesthesiologists class							0.934
I	5	1.44	2	1.16	3	1.71	
II	84	24.14	40	23.12	44	25.14	
III	236	67.82	119	68.79	117	66.86	
IV	23	6.61	12	6.94	11	6.29	
Preexisting comorbidities							
Myocardial infarction	50	14.37	27	15.61	23	13.14	0.544
Chronic heath failure	45	12.93	22	12.72	23	13.14	0.675
Atrial fibrillation	83	23.85	42	24.28	41	23.43	0.9
Valvular disease	52	14.94	26	15.03	26	14.86	1
Peripheral artery disease	40	11.49	18	10.4	22	12.57	0.615
Stroke	31	8.91	17	9.83	14	8	0.577
Diabetes	66	18.97	36	20.81	30	17.14	0.27
Hypertension	226	64.94	114	65.9	112	64	0.737
Chronic kidney disease	93	26.72	48	27.75	45	25.71	0.717
COPD	44	12.64	25	14.45	19	10.86	0.337

COPD = chronic obstructive pulmonary disease, and PMI = perioperative myocardial injury.

### PMI

PMI occurred in 23% of patients (80/348) undergoing surgery for PFF. The incidence was comparable between the arthroplasty and osteosynthesis groups (22.0% vs. 24.0%, p = 0.7) (Table II).

**TABLE II:** Overall and Group Specific Incidence of Perioperative Myocardial Infarction/Injury and Mortality

Characteristic	Total		Arthroplasty		Osteosynthesis		p
	N	%	N	%	N	%	
Total	348	100	173	49.71	175	50.29	
PMI	80	23.0	38	22.0	42	24.0	0.703
30-d mortality	20	5.7	8	4.6	12	6.9	0.491
1-yr mortality	62	17.8	27	15.6	35	20.0	0.327

PMI = perioperative myocardial infarction/injury.

Univariable logistic regression identified a medical history of myocardial infarction (odds ratio [OR] = 2.1, p = 0.02) and hypertension (OR = 1.8, p = 0.03) as significant risk factors for PMI. Among the 2 risk scores assessed, only the Revised Cardiac Risk Index was significantly associated with PMI. No associations were found between PMI and fracture-specific, hospitalization-specific, surgical, or anesthesia-related factors (Table III).

**TABLE III:** Univariable Association of Risk Factors with PMI

Characteristic	N	Odds Ratios	95% CI	p
Baseline				
Age	348	1.032	0.9936-1.073	0.111
Male	348	1.618	0.968-2.693	0.065
Height	338	1.022	0.992-1.054	0.151
BMI	338	0.9982	0.9431-1.055	0.949
Medical history of				
Myocardial infarction	348	2.141	1.111-4.035	0.02
Atrial fibrillation	348	1.287	0.7194-2.249	0.384
Peripheral artery disease	348	1.974	0.9547-3.943	0.058
Stroke	348	1.68	0.728-3.655	0.203
Hypertension	348	1.843	1.065-3.3	0.033
Chronic kidney disease	348	1.682	0.9748-2.866	0.058
COPD	348	1.683	0.8232-3.307	0.14
Preoperative hemoglobin	346	0.9988	0.9859-1.012	0.859
Risk scores				
ASA risk score (ref: 1/2)	89			0.579
3	236	1.349	0.7478-2.533	
4	23	1.495	0.4813-4.225	
Revised cardiac risk index (ref: 0)	209			<0.001
1	82	1.091	0.5577-2.059	
2	41	3.187	1.547-6.501	
3	12	4.5	1.341-15.13	
4	4	13.5	1.678-277	
Fracture specific				
Femoral neck fracture (ref: inter/subtrochanteric fracture)	179	0.8697	0.5267-1.434	0.584
Intertrochanteric fracture classification AO (ref: A1)	41			0.491
A2	80	0.6477	0.2806-1.517	
A3	48	0.587	0.2211-1.52	
Hospitalization specific				
Duration between hospital admission and operation (ref: 0-12 h)	40			0.37
12-24 h	133	0.5731	0.2603-1.305	
24-36 h	84	0.8484	0.3725-1.989	
36-48 h	51	0.6923	0.2685-1.779	
>48 h	40	0.397	0.1242-1.161	
Timepoint of start of operation (ref: 8:00-18:00)	219			0.274
18:00-24:00	47	0.5765	0.2251-1.294	
0:00-8:00	82	1.208	0.6674-2.14	
Surgery specific				
Type of implant (ref: Cephalomedullary nail)	164			0.374
Extramedullary	11	0.3	0.0161-1.638	
Hemiarthroplasty	140	0.9252	0.5443-1.564	
Total hip arthroplasty	33	0.5357	0.1733-1.373	
Type of operation				0.652
Osteosynthesis (ref: arthroplasty)	175	1.122	0.6805-1.854	
Femoral fixation in arthroplasty				
Cemented (ref: uncemented)	147	0.4174	0.09508-1.292	0.175
Operation duration from incision until wound closure	348	0.9984	0.9916-1.005	0.629
Anesthesia specific				
Type of anesthesia (ref: ETI)	260			0.738
LMA	4	0.3606	0.002707-3.449	
Spinal anesthesia	84	0.9657	0.5304-1.704	

ASA = American Society of Anesthesiologists, AO = arbeitsgemeinschaft osteosynthese; BMI = body mass index; COPD = chronic obstructive pulmonary disease; ESC = European Society of Cardiology; ETI = endotracheal intubation; PMI = perioperative myocardial infarction/injury; LMA = laryngeal mask airway.

### Mortality

Mortality for patients undergoing surgery for PPF was 5.7% at 30 days and 17.8% at 1 year. Mortality rates were comparable between the arthroplasty and osteosynthesis groups at 30 days (4.6 vs. 6.9%, p = 0.5) and at 1 year (15.6 vs. 20%, p = 0.3) (Table II). Univariable logistic regression identified body mass index (BMI) (OR = 0.9, p = 0.005) a medical history of atrial fibrillation (OR = 1.9, p = 0.04), preoperative hemoglobin (OR = 0.96, p=<0.001), and an increasing ASA score as significant risk factors for postoperative 1-year mortality.

No associations were found between 1-year mortality and fracture-specific, hospitalization-specific, or surgery-specific factors (Table IV).

**TABLE IV:** Univariable Association of Risk Factors with Mortality Within the First Year After Surgery

Characteristic	N	Odds Ratios	95% CI	p
Baseline				
Age	348	1.036	0.994-1.082	0.101
Male	348	1.824	1.041-3.184	0.034
Height	338	1.007	0.9732-1.041	0.7
BMI	338	0.9051	0.842-0.9685	0.005
Medical history of				
Myocardial infarction	348	1.366	0.63-2.768	0.405
Atrial fibrillation	348	1.851	1.006-3.333	0.043
Peripheral artery disease	348	1.397	0.5966-3.001	0.412
Stroke	348	1.389	0.531-3.235	0.469
Hypertension	348	1.536	0.8501-2.883	0.166
Chronic kidney disease	348	1.266	0.682-2.283	0.442
COPD	348	1.905	0.8896-3.869	0.083
Preoperative hemoglobin level	346	0.9627	0.9479-0.977	<0.001
Risk scores				
ASA risk score (Ref: 1/2)	89			<0.001
3	236	7.901	2.804-33.12	
4	23	15.29	3.947-76.17	
Revised Cardiac Risk Index (Ref: 0)	209			0.156
1	82	1.623	0.8242-3.124	
2	41	2.276	0.9975-4.948	
3	12	3.105	0.7879-10.54	
4	4	2.069	0.1005-16.79	
Fracture specific				
Femoral neck fracture (ref: inter/subtrochanteric fracture)	179	0.7362	0.4219-1.276	0.277
Intertrochanteric fracture classification AO (Ref: A1)	41			0.389
A2	80	0.5468	0.2196-1.374	
A3	48	0.8379	0.3173-2.21	
Hospitalization specific				
Duration between hospital admission and operation (Ref: 0-12 h)	40			0.618
12-24h	133	1.348	0.5067-4.263	
24-36 h	84	1.725	0.6214-5.607	
36-48 h	51	1.265	0.3862-4.513	
>48 h	40	2.267	0.7203-7.973	
Timepoint of start of operation (Ref: 8:00-18:00)	219			0.843
18:00-24:00	47	1.165	0.4937-2.527	
0:00-8:00	82	1.192	0.6091-2.254	
Surgery specific				
Type of implant (Ref: Cephalomedullary Nail)	164			0.034
Extramedullary	11	0.3824	0.02049-2.101	
Hemiarthroplasty	140	0.8721	0.4903-1.537	
Totalhiparthroplasty	33	0.1194	0.006598-0.5881	
Type of operation				0.285
Osteosynthesis (Ref: Arthroplasty)	175	1.352	0.7796-2.366	
Femoral fixation in arthroplasty				
Cemented (Ref: uncemented)	147	0.6684	0.1503-2.122	0.537
Operation duration from incision until wound closure	348	0.9991	0.9917-1.006	0.797
Anesthesia specific				
Type of anesthesia (Ref: ETI)	260			0.836
LMA	4	0.4868	0.00365-4.679	
Spinal anesthesia	84	0.9011	0.459-1.683	

ASA = American Society of Anesthesiologists, AO = arbeitsgemeinschaft osteosynthese, BMI = body mass index, COPD = chronic obstructive pulmonary disease, ETI = endotracheal intubation, and LMA = laryngeal mask airway.

The presence of PMI was associated with increased 1-year mortality (27.5% vs. 14.9%, p = 0.013) (Appendix 1).

**Fig. 1:**
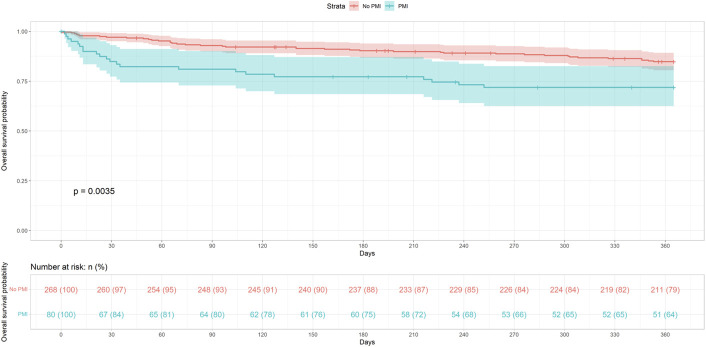
Kaplan-Meier curve showing 1-year survival stratified by the presence or absence of PMI. Survival probabilities are displayed with 95% confidence intervals for each group. PMI = perioperative myocardial injury.

The outcome of the Kaplan-Meier survival curve is shown in Fig. 1.

## Discussion

This prospective study aimed to investigate the incidence of PMI and mortality, as well as their associated orthopaedic and perioperative risk factors, following surgical treatment of PFFs at a tertiary center.

### Incidence of PMI

The incidence of PMI in our cohort of surgically treated PFFs is considerably higher than that reported for aseptic orthopaedic operations in general (23% vs. reported rates of 11.9%-14.3%^16,27^) and also surpasses that observed in the overall cohort from the BASEL-PMI study which investigated PMI rates in urology, spinal, thoracic, vascular and visceral surgery at the same institution (13.1%)^28^. We attribute this elevation to the high prevalence of comorbidities in patients with PFFs—such as coronary artery disease, heart failure, and chronic kidney disease, nonelective surgery—all of which are established independent risk factors for PMI^19-21^. Furthermore, the median age in our cohort (80 years) is higher than observed in the BASEL-PMI cohort (73 years) or in the aseptic orthopaedic cohort of the BASEL-PMI study (76 years)^28^. Several studies have demonstrated that age alone is a risk factor for PMI and that the risk of PMI increases with age^22,23^. It should be noted that the incidence of PMI in our cohort seems lower than the 35% to 40% reported in previous studies on PFF, but this likely reflects different thresholds and troponin assays used for defining PMI^19,20^.

### Mortality Rates

Overall, the 1-year mortality rate in our PFF-cohort, regardless of the occurrence of a PMI, was 17.8%. This falls within the lower range of the 1-year mortality rates reported in recent literature after surgical treatment for PFF (16.6%-29%)^29-31^. In addition, we observed a significantly higher mortality rate among patients who suffered from a PMI compared with those who did not (27.5% 1-year mortality vs. 14.9%, p = 0.013). This finding is consistent with current literature on PMI^13,32^ and adds further evidence that PMI in noncardiac surgery systematically correlates with increased mortality. As illustrated in the Kaplan-Meier curve (Fig. 1), patients with PMI in our cohort exhibited a markedly higher mortality rate during the first 5 weeks postoperatively. Beyond this period, the mortality rate in the PMI group appeared to align with that of patients who did not experience a PMI. This is consistent with previous reports investigating myocardial injury/infarction after hip surgery^32,33^.

It is essential to detect and screen patients at risk for PMI to be able to adapt postoperative care. Several guidelines^34,35^ suggest that patients identified as high risk (elevated cardiac biomarkers at hospital arrival, history of cardiovascular disease, age >65 years, and comorbidities such as chronic kidney disease and hypertension) should be incorporated into a screening and response system for PMI. The criteria applied in our institution's screening and response system are in line with those suggested by the European Society of Cardiology from 2022^35^ to improve PMI detection and optimize perioperative management.

### Risk Factors for PMI

Among the examined risk factors, a history of myocardial infarction and hypertension was the only medical comorbidities significantly associated with PMI in our cohort. This association is already well established in the literature^24,36^. Further analysis of orthopaedic, fracture-related, hospitalization, surgical, and anesthesia-specific factors revealed no association with the occurrence of PMI.

Comparable rates of PMI and mortality were observed between patients treated with arthroplasty (22%) and those treated with osteosynthesis (24%). The choice of surgical method is primarily determined by the type of fracture encountered and the functional level of the patient. To date, there are no specific data in the literature directly comparing PMI rates between arthroplasty and osteosynthesis in the context of PFF.

Our results showed no association between surgical delay and PMI. However, a substudy of the HIP ATTACK trial found that accelerated surgery in patients with elevated admission troponin reduced mortality, major complications, and postoperative MI compared with standard care^37^. The accelerated group also had fewer cases of recurrent myocardial injury without ECG changes or symptoms, aligning with the definition of PMI.

### Risk Factors for Mortality

Among the examined risk factors low BMI, a history of atrial fibrillation, low preoperative hemoglobin, and the use of THA showed to be significantly associated with 1-year mortality in our cohort.

We observed a significantly lower 1-year mortality rate among patients receiving THA (OR = 0.12, p = 0.034). Similarly, a large prospective cohort of nearly 25,000 patients reported lower 120-day mortality after THA (2%) compared with CMN (11.2%) and hemiarthroplasty (13.6%)^38^. As in our cohort, this likely reflects selection bias, as healthier, younger patients are more often chosen for THA, while older or more comorbid patients typically receive hemiarthroplasty

When comparing the use of CMN with hemiarthroplasty for intertrochanteric fracture, there is some evidence for a potential advantage in terms of lower mortality for patients treated with CMN^39-41^. Nevertheless, the results of our investigation do not support this potential advantage, with a similar 1-year mortality in both groups.

Our findings indicated that a surgical delay of more than 48 hours was associated with a 2.3-fold increase in the odds of 1-year mortality compared with patients who underwent surgery within 12 hours. This finding aligns with Leer-Salvesen et al., who analyzed over 70,000 proximal femoral fractures in the Norwegian Hip Fracture Register, and found no increase in mortality or intraoperative complications within 48 hours, but a significant rise in mortality with delays beyond this period^42^.

### Limitations

Given that troponin levels were systematically measured in all patients in our cohort and PMI were therefore identified and potentially treated, the overall 1-year mortality rate is potentially lower than in an unscreened population. This may have led to an underestimation of the true impact of PMI on 1-year mortality. In addition, because surgical treatment in PFF is largely determined by fracture location, fixation method and fracture type cannot be considered 2 independent risk factors. Finally, the small sample size in the cemented femoral fixation group limited our ability to conduct a detailed analysis of the association between cementation and PMI rates.

## Conclusions

Patients undergoing surgical PFF treatment are at high risk of PMI, and the occurrence of PMI is correlated with increased mortality. The type of implant and the use of bone cement for PFF treatment are not related to increased PMI rate. Surgical delay of more than 48 hours is associated with increased mortality, but not with increased PMI rate. We recommend systematic screening of patients scheduled to undergo surgery for PFF to improve PMI detection and optimize perioperative management.

## Funding

This work was supported by the Swiss National Science foundation (179362, C. Müller); the Swiss Heart Foundation (C. Müller, C. Puelacher); Roche Diagnostics (C. Puelacher, C. Müller).

## Author Contributions

M. Wittauer: conceptualization, data curation, investigation, methodology, project administration, writing—original draft, writing—review & editing. M-A. Burch: data curation, investigation, writing—original draft, writing—review & editing. C. Puelacher: conceptualization, funding acquisition, methodology, validation, writing—review & editing. F. Halbeisen: data curation, formal analysis, validation. M. Clauss: resources, supervision, writing—review & editing. A. Müller: funding acquisition, resources, writing—review & editing. C. Müller: conceptualization, funding acquisition, methodology, resources, writing—review & editing. M. Morgenstern: conceptualization, investigation, methodology, project administration, supervision, Writing—review & editing.

## Appendix

Supporting material provided by the authors is posted with the online version of this article as a data supplement at jbjs.org (http://links.lww.com/JBJSOA/B61). This content was not copyedited or verified by JBJS.
